# An ImmunoPEGliposome for Targeted Antimalarial Combination Therapy at the Nanoscale

**DOI:** 10.3390/pharmaceutics11070341

**Published:** 2019-07-16

**Authors:** Arnau Biosca, Lorin Dirscherl, Ernest Moles, Santiago Imperial, Xavier Fernàndez-Busquets

**Affiliations:** 1 Barcelona Institute for Global Health (ISGlobal, Hospital Clínic-Universitat de Barcelona), Rosselló 149-153, ES-08036 Barcelona, Spain; 2 Nanomalaria Group, Institute for Bioengineering of Catalonia (IBEC), The Barcelona Institute of Science and Technology, Baldiri Reixac 10-12, ES-08028 Barcelona, Spain; 3 Nanoscience and Nanotechnology Institute (IN2UB), University of Barcelona, Martí i Franquès 1, ES-08028 Barcelona, Spain; 4 Children’s Cancer Institute, Lowy Cancer Research Centre, UNSW Sydney, PO Box 81, Randwick, NSW 2031, Australia; 5 School of Women’s and Children’s Health, UNSW Sydney, Sydney, NSW 2052, Australia; 6 Department of Biochemistry and Molecular Biomedicine, University of Barcelona, Avda. Diagonal 643, ES-08028 Barcelona, Spain

**Keywords:** combination therapy, immunoliposomes, malaria, nanomedicine, nanotechnology, *Plasmodium*, targeted drug delivery

## Abstract

Combination therapies, where two drugs acting through different mechanisms are administered simultaneously, are one of the most efficient approaches currently used to treat malaria infections. However, the different pharmacokinetic profiles often exhibited by the combined drugs tend to decrease treatment efficacy as the compounds are usually eliminated from the circulation at different rates. To circumvent this obstacle, we have engineered an immunoliposomal nanovector encapsulating hydrophilic and lipophilic compounds in its lumen and lipid bilayer, respectively. The antimalarial domiphen bromide has been encapsulated in the liposome membrane with good efficiency, although its high IC_50_ of ca. 1 µM for living parasites complicates its use as immunoliposomal therapy due to erythrocyte agglutination. The conjugation of antibodies against glycophorin A targeted the nanocarriers to *Plasmodium*-infected red blood cells and to gametocytes, the sole malaria parasite stage responsible for the transmission from the human to the mosquito vector. The antimalarials pyronaridine and atovaquone, which block the development of gametocytes, have been co-encapsulated in glycophorin A-targeted immunoliposomes. The co-immunoliposomized drugs have activities significantly higher than their free forms when tested in in vitro *Plasmodium falciparum* cultures: Pyronaridine and atovaquone concentrations that, when encapsulated in immunoliposomes, resulted in a 50% inhibition of parasite growth had no effect on the viability of the pathogen when used as free drugs.

## 1. Introduction

Despite the undeniable importance of malaria elimination on the global research agenda, current vaccines in development do not offer prospects of complete protection [1] and the available drugs are rapidly losing efficacy, with resistance already evolved to the front-line drug artemisinin [2]. This scenario has led to the continuing need of research for new antimalarial agents [3] and to the use of combinations of drugs that do not share the same resistance mechanisms [4,5,6]. However, the different pharmacokinetic profiles often exhibited by the combined drugs tend to decrease treatment efficacy as the compounds are usually eliminated from the circulation at different rates [7]. Encapsulation of drugs in targeted nanovectors is a rapidly growing area with a clear applicability to infectious disease treatment [8], and pharmaceutical nanotechnology has been identified as a potentially essential tool in the future fight against malaria [9,10]. Liposomes (LPs) in particular are an ideal platform to develop drug delivery systems for combination therapies at the nanoscale, due to their capacity to incorporate in a single nanostructure molecules with widely diverging characteristics (and antimicrobial mechanisms), namely, hydrophilic drugs in their aqueous core and lipophilic compounds in their lipid bilayer.

In all organisms studied so far, isopentenyl diphosphate (IPP) is a key intermediate of isoprenoid biosynthesis [11]. In mammals and fungi, IPP is derived from the mevalonate pathway. In about 1993, the 2-*C*-methyl-d-erythritol-4-phosphate (MEP) pathway was identified as an alternative route for the early steps in the biosynthesis of isoprenoids [12,13]. Whereas the MEP pathway is absent in mammals, it is essential for many human pathogens, including *Plasmodium* [14], thus its enzymes are attractive targets for the development of novel antimalarials. This metabolic route begins with the condensation of glyceraldehyde-3-phosphate and pyruvate to yield 1-deoxy-d-xylulose 5-phosphate (DOXP), which is subsequently converted to MEP by the enzyme DOXP reductoisomerase (DXR) [15]. The MEP pathway has been identified in the apicoplast, a relic chloroplast of *Plasmodium* and related protists, where it plays an essential function for the pathogen’s survival [16,17], and, since it has not been extensively used yet as a target to treat malaria, it is expected that the parasite will not show significant resistance against inhibitors of its enzymes. The drug fosmidomycin inhibits the recombinant DXR from *Plasmodium falciparum* with an IC_50_ value of ~28 nM and shows activity on the intact parasite, both in in vitro *P. falciparum* cultures (IC_50_ between 290 and 370 nM depending on the parasite strain) and in in vivo assays with mice infected with the rodent malaria species *Plasmodium vinckei* [16]. In preliminary clinical trials fosmidomycin had been shown to be efficacious for the treatment of malaria in children [18], although a high rate of relapse is complicating its use as a monotherapy [19]. Presumably, compounds inhibiting other MEP pathway steps will also show antiplasmodial activity. Fluoropyruvate, for instance, has been described as an inhibitor of bacterial DOXP synthase [20], and is currently being studied as a potential new antimalarial [21]. The Malaria Box compound MMV008138 targets the third enzyme of the MEP pathway, IspD, which catalyzes the formation of 4-diphosphocytidyl-2-*C*-methyl-d-erythritol [22]. The quaternary ammonium compound domiphen bromide (DB) has also been shown in in vitro enzyme activity assays to inhibit IspD from *Mycobacterium smegmatis* [23] and from *Plasmodium vivax* [24].

Preexisting data indicate that the lipids used to assemble LPs targeted to *Plasmodium*-infected red blood cells (pRBCs) were incorporated by the parasites [25]. The molecular dimensions of DB suggest that it can be inserted into biological membranes to constitute a liposoluble drug cargo in a LP designed for the targeted delivery of antimalarial combination therapies. As a potential partner drug for such nanovessel, the water-soluble compound pyronaridine has shown high potency against *P. falciparum*, including chloroquine-resistant strains [26]. Although several mechanisms of action have been described for pyronaridine [26], its main target seems to be related to haem detoxification in the food vacuole of the parasite. Resistance to pyronaridine appears to emerge slowly and is further retarded when it is used in combination with other antimalarials, being its toxicity generally less than that of chloroquine. The recent appreciation that efficient antimalarial strategies will require the interruption of parasite transmission from the human host to the vector [27] has prompted the search for transmission-blocking drugs [28]. One target of such drugs is the gametocyte, which is the parasite stage solely responsible for *Plasmodium* transmission to the mosquito. Pyronaridine exhibits a moderate inhibition of early gametocyte stages and is one of the few drugs active against mature gametocyte transmission to *Anopheles* mosquitoes [29,30,31], which makes it an interesting compound for combination therapies [29].

**Figure 1:**
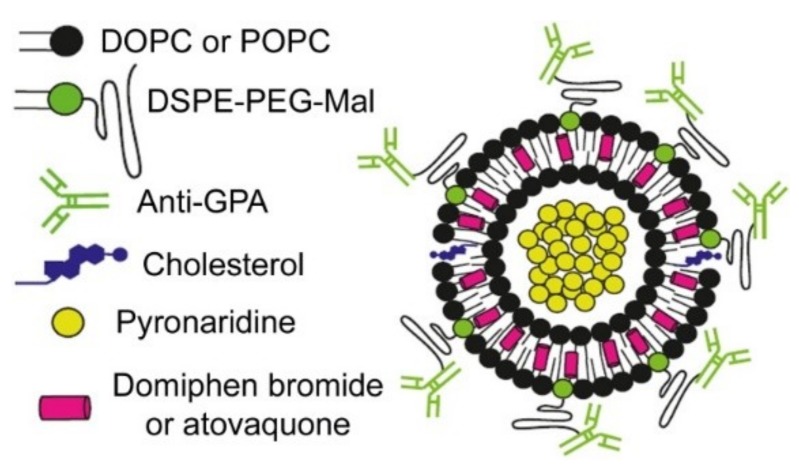
Scheme of the immunoliposome designed here for targeted antimalarial combination therapy.

In addition to carrying the active ingredients, an optimal nanovector should be designed to have specificity towards the cell type where therapeutic activity has to be unleashed. Using glycophorin A (GPA) as an immunoliposomal target present in both non-parasitized red blood cells (RBCs) and in pRBCs, the efficacy of drugs encapsulated in LPs could be significantly improved due to the prophylactic effect of loading antimalarial compounds into RBCs when these have not yet been infected by *Plasmodium* [32,33,34]. Since GPA is also present in the gametocyte membrane, it is an ideal receptor for targeting strategies against all blood stages of the malaria parasite. Here we have integrated the strategies outlined above into the design of a GPA-targeted immunoliposome (iLP, Figure 1) encapsulating in its aqueous lumen pyronaridine and in its lipid bilayer DB or the lipophilic antimalarial drug atovaquone (whose site of action is complex III of the mitochondrial respiratory chain [35]), with the objective of exploring the capacity of nanocarriers to be developed into new antimalarial combination therapies at the nanoscale.

## 2. Materials and Methods

### 2.1. Materials

Except where otherwise indicated, reagents were purchased from Sigma-Aldrich Corporation (St. Louis, MO, USA), and reactions were performed at room temperature (22 to 24 °C). The lipids (all ≥ 99% purity according to thin layer chromatography analysis) 1,2-dioleoyl-*sn*-glycero-3-phosphocholine (DOPC), 1-palmitoyl-2-oleoyl-glycero-3-phosphocholine (POPC), 1,2-distearoyl-*sn*-glycero-3-phosphocholine (DSPC), 1,2-distearoyl-*sn*-glycero-3-phosphoethanolamine-N-[methoxy(polyethylene glycol)-2000] (DSPE-PEG2000), 1,2-dioleoyl-*sn*-glycero-3-phosphoethanolamine-N-(lissamine rhodamine B sulfonyl) (DOPE-Rho) and 1,2-distearoyl-*sn*-glycero-3-phosphoethanolamine-N-[maleimide(polyethylene glycol)-2000] (DSPE-PEG2000-Mal) were purchased from Avanti Polar Lipids Inc. (Alabaster, AL, USA) and stored at −20 °C. Monoclonal HIR2 antibodies raised against the N-terminal portion of GPA were obtained from Acris Antibodies GmbH (Herford, Germany).

### 2.2. Generation of Liposomes

Liposomes were prepared by the thin lipid film hydration method [36] (Figure S1). The lipid formulations DOPC/POPC/DSPC:DSPE-PEG2000:cholesterol 75:5:20 (DOPC-, POPC-, and DSPC-based liposomes, respectively) were obtained by mixing stock solutions of lipids in chloroform:methanol (2:1 *v/v*) in a round bottom flask. When required for antibody binding, DSPE-PEG2000-Mal was substituted for DSPE-PEG2000; when required for liposome labeling, 0.5% DOPE-Rho was incorporated in the formulation, simultaneously reducing the DOPC, POPC, or DSPC content to 74.5%. Organic solvents were removed by rotatory evaporation (RS 3000-V rotatory evaporator, Selecta) at 37 °C under reduced pressure. The thin lipid film formed on the flask walls was hydrated in phosphate buffered saline (PBS, pH 7.4) or in 200 mM citrate buffer, pH 3.5, in a volume corresponding to a final lipid concentration of 10 mM (unless otherwise specified all lipid concentrations refer to total lipid including cholesterol). Mostly unilamellar LPs were obtained by 3 rounds of constant vortexing for 2 min followed by bath sonication at 35 °C for 3 min (FB15053 ultrasonic bath, Thermo Fisher Scientific, Inc., Waltham, MA, USA), and by extrusion through 400 nm polycarbonate membranes (Avanti Polar Lipids, Inc., Alabaster, AL, USA) using a mini extruder device (Avanti Polar Lipids, Inc., Alabaster, AL, USA). Throughout the lipid film hydration and downsizing processes, samples were maintained above the lipids’ transition temperature. Sterility of LP samples was maintained by rinsing all material in 70% ethanol and working in a laminar flow hood. After each preparation all materials were immersed in 70% ethanol, sonicated for 15 min at 60 °C, and, finally, thoroughly rinsed with double-distilled water (ddH_2_O, MilliQ system, Millipore, Burlington, MA, USA). Dynamic light scattering size and polydispersity measurement of LPs was done after 1:30 sample dilution in PBS, using a Zetasizer NanoZS90 (Malvern Ltd., Malvern, UK).

### 2.3. Encapsulation of Drugs in Liposomes

DOPC-based LPs containing DB in their membrane were generated by the thin lipid film hydration method, adding the desired amount of a 50 mM DB stock solution in methanol to the initial lipid composition dissolved in chloroform:methanol (2:1 *v/v*) to form LPs with molar ratios lipid:DB of 10:1, 10:2.5, and 10:5. In preliminary assays, liposome preparations containing DB in their lipid bilayers were examined using different lipids as the main formulation component. Whereas DSPC (saturated)-based liposomes resulted in large aggregates (Figure S2A), DOPC (unsaturated)-based liposome suspensions consisted of vesicles with a mean diameter around 180 nm (Figure S2B,C) and a polydispersity index of 0.36.

POPC-based LPs containing atovaquone in their membrane were also generated by the thin lipid film hydration method, adding the desired amount of a 1 mM atovaquone stock solution in methanol to the initial lipid composition dissolved in chloroform:methanol (2:1 *v/v*) to form LPs with molar ratios lipid:atovaquone of 10:0.125, 10:0.5, and 10:1. In preliminary assays, liposome preparations containing atovaquone in their lipid bilayers were examined using different lipids as the main formulation component. Whereas DOPC-based liposomes resulted in a poorer atovaquone encapsulation, as after 24 h a yellow precipitate (free atovaquone) was observed, POPC-based LPs offered a more stable preparation, without any observable precipitate (data not shown).

For pyronaridine encapsulation, unilamellar LPs were formed in 200 mM citrate buffer, pH 3.5, which was exchanged with PBS at pH 7.4 using 7-kDa Zeba^TM^ spin desalting columns (Thermo Fisher Scientific, Inc.), creating a pH gradient between the outside and the inside of the LPs (Figure S1). In order to avoid osmotic rupture of the LPs during buffer exchange, PBS was made isotonic with the citrate buffer by addition of NaCl while measuring osmolarity with an Advanced Instruments^TM^ Micro-Sample Model 3320 Osmometer (Thermo Fisher Scientific, Inc.). To these pH gradient-holding LPs (containing 10 mM lipid) was added one volume of 2× concentrated pyronaridine solution in PBS, and this mixture was kept at room temperature for 17 h before drug encapsulation was quantified (see below). Non-encapsulated pyronaridine was removed by pelleting liposomes by ultracentrifugation (150,000× *g*, 2.5 h, 4 °C) and substituting the supernatant by isotonic PBS. Drug-encapsulating LPs were stored at 4 °C for up to 14 days.

### 2.4. Generation of Immunoliposomes

Immunoliposomes were obtained by coupling anti-GPA antibody to the maleimide group in DSPE-PEG2000-Mal following established protocols [32] (Figure S3), which resulted in the incorporation, on average, of ca. 85 antibody molecules/liposome. Briefly, anti-GPA stock solutions (1 mg/mL) were buffer exchanged with PBS and incubated at room temperature for 30 min with N-succinimidyl-S-acetylthioacetate crosslinker (SATA, Thermo Fisher Scientific, Inc.) at a molar ratio of anti-GPA:SATA 1:10; unreacted SATA was removed by buffer exchange with PBS. Thioester groups on the anti-GPA-bound SATA were deacetylated through addition at room temperature of 0.1 volume of 500 mM hydroxylamine, 25 mM EDTA in PBS. After deacetylation, thiolated anti-GPA was buffer exchanged with osmolarity-adjusted PBS buffer supplemented with 10 mM EDTA. Maleimide-containing LPs were coupled to thiolated anti-GPA by incubation for 17 h at room temperature. For pyronaridine-encapsulating iLPs, pyronaridine solution was added 30 min before the anti-GPA solution and the mixture was also incubated for 17 h at room temperature. Free anti-GPA and pyronaridine were finally removed by ultracentrifugation (150,000× *g*, 2.5 h, 4 °C).

### 2.5. Quantification of Encapsulated Drugs

Liposomes encapsulating (i) pyronaridine, (ii) DB, or (iii) atovaquone were disrupted by treatment with, respectively, (i) one volume of 4% SDS, (ii) 3 volumes of DMSO, or (iii) 3 volumes of lysis buffer (2.66% SDS, 0.166 mM NaOH and 33% DMSO in H_2_O). Drugs present in the resulting solutions were quantitated by absorbance spectroscopy (Epoch^TM^ microplate spectrophotometer, BioTek Instruments, Inc., Winooski, VT, USA), measuring A_426nm_ for pyronaridine, A_270nm_ for DB, and A_278nm_ for atovaquone; the corresponding absorbances of drug-free liposomes were subtracted. Encapsulation efficiencies are defined as the fraction of encapsulated drug relative to the initially added concentration.

### 2.6. P. falciparum Cultures

The 3D7 *P. falciparum* strain was cultivated in blood group B human RBCs (3% hematocrit) following standard protocols [37]. The human blood used in this work was commercially obtained from the *Banc de Sang i Teixits* (www.bancsang.net). Blood was not specifically collected for this research; the purchased units had been discarded for transfusion, usually because of an excess of blood relative to anticoagulant solution. Prior to their use, blood units underwent the analytical checks specified in the current legislation. Before being delivered to us, unit data were anonymized and irreversibly dissociated, and any identification tag or label had been removed in order to guarantee the non-identification of the blood donor. No blood data were or will be supplied, in accordance with the current Spanish *Ley Orgánica de Protección de Datos* and *Ley de Investigación Biomédica*. The blood samples will not be used for studies other than those made explicit in this research. The studies reported here were performed under protocols reviewed and approved by the Ethical Committee on Clinical Research from the *Hospital Clínic de Barcelona* (Reg. HCB/2014/0910, October 14, 2014).

Parasites (thawed from glycerol stocks) were grown at 37 °C in Roswell Park Memorial Institute complete medium containing AlbuMAX^®^ II (RPMI-A, Gibco, Glasgow, UK) under a gas mixture of 92% N_2_, 5% CO_2_, and 3% O_2_. *P. falciparum*-infected erythrocytes were identified by staining their nuclei for 10 min with Giemsa (Merck Chemicals, Darmstadt, Germany) diluted 1:10 in Sorenson’s buffer, pH 7.2, and microscopically counted in blood smears fixed with methanol. For culture maintenance, parasitemia was kept below 5% late forms and 10% early forms by dilution with freshly washed RBCs and RPMI-A was changed every 1–2 days. Cultures were synchronized in early ring stages (0–24 h post-invasion) by 5% sorbitol lysis [38]. Gametocytes were obtained using the gametocyte-generating E5 subclone of the 3D7 strain, kindly provided by Dr. Alfred Cortés. Briefly, a culture containing 10% rings was supplemented with 50 mM N-acetylglucosamine to inhibit asexual replication and select those sexually committed ring stage parasites. After 24 h the medium was replaced daily for 2 weeks with RPMI-A containing 50 mM N-acetylglucosamine, without further adding fresh blood.

### 2.7. Growth Inhibition Assays of P. falciparum Blood Stages

In vitro growth inhibition assays were conducted using synchronized *P. falciparum* cultures (>95%) in the early ring stage at 4% hematocrit and 1.5% parasitemia as previously described [32]. Unless otherwise specified, one culture volume of 2× concentrated drug solution in RPMI-A was added to the parasitized cell suspension, and cultures were incubated under orbital stirring in Petri dishes. After 15 min, cultures were transferred to microcentrifuge tubes and washed 3× (5 min, 300× *g*) with fresh RPMI-A, incubating for 15 min between washes. Prior to each wash and after the last wash, triplicates of small volume fractions of cultures were seeded in a 96-well plate and further incubated for a complete 48 h growth cycle under the conditions described above. For the determination of parasitemia, samples were diluted 1:100 in isotonic PBS and the nuclei of pRBCs (the only nucleated cells present in the culture) were stained by addition of 0.1 μM Syto11 (Thermo Fisher Scientific, Inc.) in the final mixture before proceeding to flow cytometry analysis (see below). Growth inhibition in drug-treated samples was defined as the percentage decrease in parasitemia within the second generation of parasites relative to untreated control samples. Growth inhibition graphs and IC_50_ values were obtained through sigmoidal fitting of growth data at different drug concentrations using the GraphPad Prism Software version 6.00 (www.graphpad.com; San Diego, CA, USA). For IC_50_ calculation, a Sigmoidal 4PL curve fitting model was used (fixed top = 100, fixed bottom = 0, confidence interval = 95%), where X was the logarithm of drug concentration and Y was the percentage of growth inhibition relative to untreated control samples.

### 2.8. Cell Binding Assays

Different solutions of decreasing iLP and LP concentrations were obtained through serial dilution in RPMI-A, mixed with one sample volume of a RBC or pRBC culture prepared at 6% hematocrit (and 3% parasitemia in the case of pRBC assays), and incubated (15 min, 23 °C, 700 rpm) in a Thermomixer comfort (Eppendorf AG, Hamburg, Germany). Prior to analysis, samples were diluted 1:100 in PBS and centrifuged for 5 min at 300× *g*, except for flow cytometry analysis (see below), where cells were pelleted at 100× *g*. Stock solutions not undergoing centrifugation and supernatants were analyzed by fluorescence spectroscopy measurement of DOPE-Rho incorporated in the LP formulations (Synergy^TM^ HT multidetection microplate reader, BioTek Instruments, Inc.) with a 530/25 nm excitation filter and a 645/40 nm emission filter. Fluorescence readings were carried out in duplicates.

### 2.9. Liposome Stability and Drug Release Assays

For stability assays, LPs containing pyronaridine were stored at 4 °C and at different timepoints encapsulated pyronaridine was determined by absorption spectroscopy, as described above. For drug release assays LPs containing pyronaridine were ultracentrifuged (150,000× *g*, 2.5 h, 4 °C) and pellets were resuspended in RPMI-A, pRBC or RBC cultures, or osmolarity-adjusted PBS to obtain a liposome suspension containing 5 mM lipid. In order to simulate the conditions in *P. falciparum* cultures, LPs resuspended in RPMI-A were mixed in a Thermomixer comfort (15 min, 23 °C, 700 rpm) and subsequently warmed in a PSC96 Thermoblock (Grant Instruments Ltd., Cambridge, UK) at 37 °C. At different timepoints liposomes were pulled down (150,000× *g*, 45 min, 4 °C) and pyronaridine concentration in the supernatants was determined by absorption spectroscopy as described above. Two additional control samples were included: Sonicated LPs containing pyronaridine (65 °C, 20 min sonication) and LPs containing pyronaridine treated with one sample volume of 4% SDS. Drug release was defined as the percentage of pyronaridine found in the supernatant relative to the initially encapsulated drug.

### 2.10. Flow Cytometry

For targeting and growth inhibition assays, samples were analyzed at 0.02% hematocrit in PBS with a BD LSRFortessa^TM^ cell sorter (Becton, Dickinson and Company, Franklin Lakes, NJ, USA). Forward- and side-scatter areas in a linear scale were used to gate the RBC population. Syto11-stained pRBCs were detected by excitation through a 488 nm laser at 50 mW power and emission collection with a 530/30 nm bandpass filter in logarithmic scale. RBCs targeted by DOPE-Rho-containing anti-GPA iLPs were detected by excitation through a yellow–green 561 nm laser at 50 mW power and emission collection with a 610/20 nm bandpass filter and a 600 nm long pass dichroic mirror (PE-TRED-YG-channel) in logarithmic scale. Acquisition was configured to stop after recording ≥ 10,000 events in the RBC population. In order to accurately determine the parasitemia of cultures by flow cytometry, only singlets were used. RBC agglutinates were removed from the analysis by discarding cytometer events with a disproportion on the forward-scattered light height-area (H-A) confronted parameters, which indicated the presence of cell doublets and agglutinates as previously described [32].

### 2.11. Fluorescence Microscopy

For microscopy targeting analysis, pRBCs present in the samples were stained by incubation with 0.2 μg/mL Hoechst 33342 (Molecular Probes) and placed into a Nunc™ Lab-Tek™ chambered coverglass prior to image acquisition with an Olympus IX51 inverted system microscope, equipped with an IX2-SFR X-Y stage, a U-TVIX 2 camera, and a fluorescence mirror unit cassette for UV/blue/green excitation and detection of their respective blue/green/red emission ranges.

### 2.12. Cryogenic Transmission Electron Microscopy (cryoTEM)

After glow-discharge to make the carbon film on a Holey Carbon 400-mesh copper grid hydrophilic, a 3-μL drop of the sample was deposited onto it. The grid was mounted on an EM GP plunger (Leica Microsystems GmbH, Wetzlar, Germany) and blotted with a Whatman No. 1 filter paper. The LP dispersion was immediately vitrified by rapid immersion in liquid ethane. The grid with the vitrified sample was mounted on a Gatan 626 cryo-transfer system and inserted into a Jeol JEM 2011 cryo-electron microscope operated at 200 kV, under low-dose conditions, and using different degrees of defocus (500–900 nm) to obtain an adequate phase contrast. Images were recorded on a Gatan Ultrascan US1000 CCD camera and analyzed with the Digital Micrograph 1.8 software.

## 3. Results

### 3.1. Liposome Encapsulation of Pyronaridine

**Figure 2:**
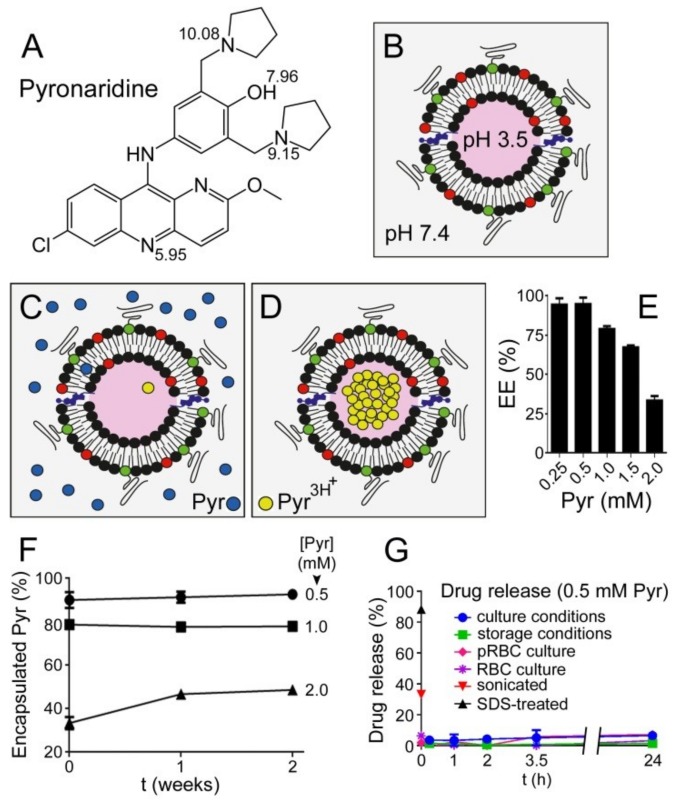
Pyronaridine encapsulation. (**A**) Pyronaridine structure with the pKas of relevant protonable groups indicated. (**B**) Scheme of the transmembrane 7.4→3.5 pH gradient. (**C**,**D**) Scheme of pyronaridine (Pyr) encapsulation. (**E**) Pyronaridine encapsulation efficiency (EE) in LPs (5 mM lipid) at different initial drug concentrations. (**F**) Stability of pyronaridine encapsulation at 4 °C. (**G**) Pyronaridine release assay from LPs (molar ratio lipid:pyronaridine ca. 10:1) incubated under different conditions (see text for details).

Depending on the pH, pyronaridine (Figure 2A) can be present in different ionization forms according to the pKas of its hydroxyl group and three protonable amino groups [39]. At the blood pH of 7.4 pyronaridine is relatively lipophilic (logD 0.34) [26] and, therefore, a significant fraction of it will likely interact with lipid bilayers, whereas at lower pH lipophilicity is reduced due to the appearance of fully protonated forms (at pH 3.5, 99.6% of pyronaridine molecules are protonated in all three amino groups [39]). When LPs are generated holding a pH gradient from 7.4 outside to 3.5 inside the vesicle (Figure 2B), uncharged pyronaridine molecules present in the LP suspension will be able to enter the liposomal lipid bilayer; there, those molecules eventually facing the luminal part of the vesicles will be exposed to the intraliposomal acidic pH and protonated, leaving the membrane and becoming encapsulated (Figure 2C,D). The encapsulation efficiency of pyronaridine incubated for 17 h with such a pH gradient-sustaining LP suspension containing 5 mM lipid was >95% up to 0.5 mM drug (Figure 2E), corresponding to a lipid:pyronaridine molar ratio close to 10:1. At higher pyronaridine concentrations (1, 1.5, and 2 mM) the encapsulated fraction was proportionally lower (ca. 80%, 70%, and 35%, respectively). The requirement for a correctly-oriented pH gradient is evidenced in controls where the absence of a gradient or its inversion resulted in the lack of pyronaridine encapsulation (Figure S4).

As pyronaridine becomes entrapped inside LPs, the internal proton pool is progressively depleted, until eventually cancelling the pH gradient. To investigate if this pH gradient exhaustion led to drug leakage, the amount of encapsulated pyronaridine in the samples from Figure 2E was followed over a storage time of two weeks at 4 °C. At the starting concentrations of 0.5 and 1 mM drug, the percentage of encapsulation was maintained relatively stable at about 90% and 80%, respectively, for the duration of the assay (Figure 2F). At 2 mM pyronaridine the encapsulated drug was observed to increase during the first week from ca. 35% to ca. 45%, indicating that after 17 h of incubation the LPs had not reached their full encapsulation capacity. Since no drug leakage was observed over two weeks, we conclude that pyronaridine uptake does not destabilize the LPs and that the pH gradient is preserved at least up to the lipid:pyronaridine ratio of 10:4 (5 mM:2 mM).

Those LPs exhibiting the highest encapsulation efficiency (ca. 10:1 lipid:pyronaridine ratio) were selected for a drug release assay (Figure 2G). After removing the non-encapsulated drug, the samples were incubated in culture medium (either in the absence or in the presence of RBCs and pRBCs) at 37 °C for 24 h, after which time only minor drug release was observed (≤6.5 ± 1.2%). Similar assays performed in storage conditions (incubation in PBS at 4 °C) resulted in even lower drug release (ca. 1% after 24 h), presumably due to the reduced fluidity and, therefore, higher impermeability of the LP lipid bilayer at this low temperature [40]. Control LPs treated with 2% SDS exhibited an immediate drug release of around 90%, in agreement with the lipid bilayer solubilization induced by the detergent [41]. Finally, somewhat surprisingly, LPs subjected to sonication released only about 35% of encapsulated pyronaridine.

### 3.2. Immunoliposome Targeting

**Figure 3:**
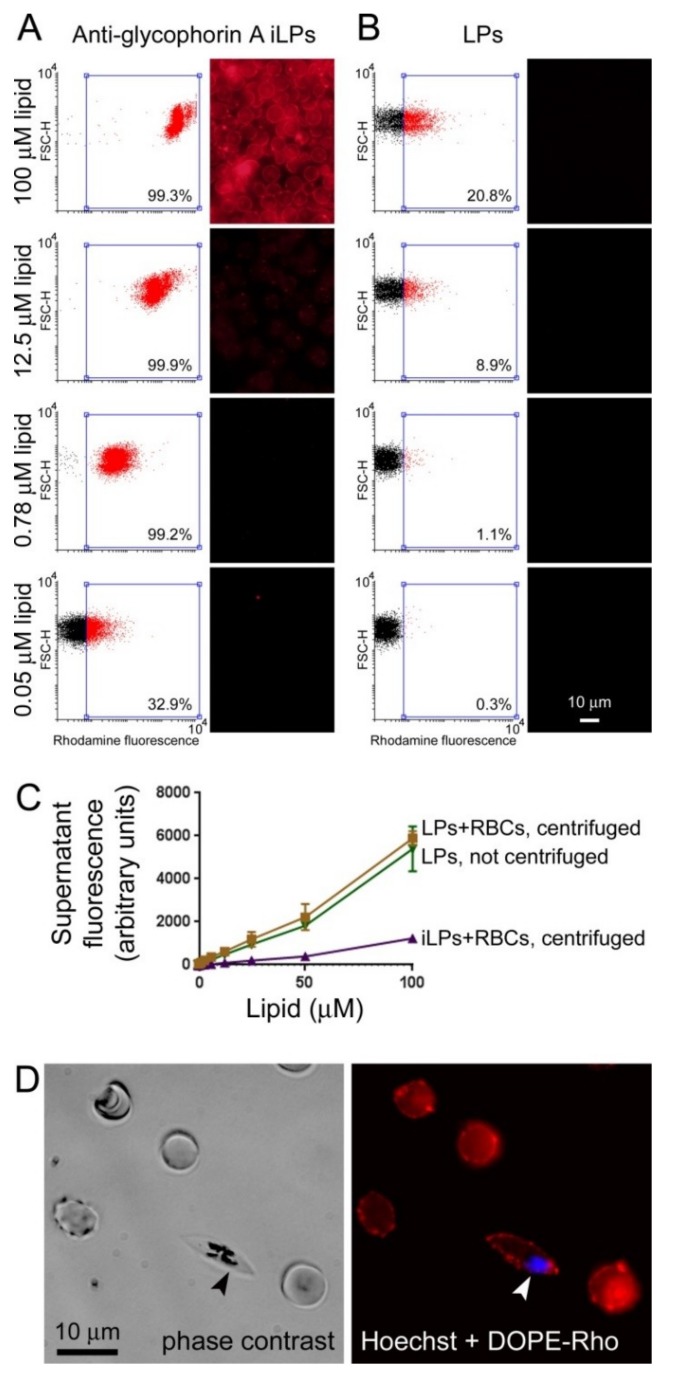
Immunoliposome targeting. (**A**,**B**) Flow cytometry and fluorescence microscopy RBC targeting analysis of DOPE-Rho-labeled (**A**) anti-glycophorin A iLPs and (**B**) plain liposomes at different (i)LP (expressed as lipid concentration):cell ratios. In each flow cytometry plot the fraction of targeted RBCs is indicated. The fluorescence microscopy settings were adjusted for a correct exposure of the 100 µM lipid iLP sample. (**C**) Fluorescence analysis of the supernatants of centrifuged samples where DOPE-Rho LPs and iLPs had been preincubated with RBCs. (**D**) Fluorescence microscopy targeting analysis of the iLP sample to *P. falciparum* gametocytes. The arrowhead indicates a gametocyte.

Pyronaridine-loaded LPs (ca. 10:1 lipid:drug ratio) targeted with antibodies against the erythrocyte membrane protein glycophorin A and labeled through the incorporation of 0.5% DOPE-Rho in their formulation were prepared following established protocols [32]. Different dilutions of these immunoliposomes were incubated with erythrocytes (3% hematocrit) and cell targeting was analyzed by flow cytometry and fluorescence microscopy (Figure 3A). At a lipid concentration of 0.78 µM, >99% RBC targeting by iLPs was achieved according to flow cytometry data, whereas only ~1% of cells were associated with LPs not bearing antibody (Figure 3B). At 100 µM lipid ca. 21% of RBCs were targeted by plain LPs; this result was not unexpected at such high liposome concentrations, and illustrates the well-known capacity of liposomes to improve cargo delivery to cells. The stronger signal in fluorescence microscopy images as the iLP concentration increased reflected the successive binding of a higher number of iLPs on the cell surface. At 0.05 µM lipid about 33% of RBCs were still being targeted by iLPs according to the threshold rhodamine signal defined by a LP-free erythrocyte control. To confirm the strong binding of iLPs to target cells, RBCs (3% hematocrit) were incubated with different rhodamine-labeled LP and iLP amounts and after 15 min of incubation the samples were spun down at a centrifuge force (300× *g*) sufficient to pellet RBCs but not LPs and iLPs. When the fluorescence of the supernatants was determined it could be concluded that virtually all non-targeted LPs remained in suspension whereas almost all iLPs were found in the cell pellet (Figure 3C). This result indicated that iLP constituents remained bound to target cells in the presence of strong forces comparable or even stronger than those encountered by RBCs in the blood circulation [42]. Gametocytes, which expose glycophorin A on their membranes, were also targeted by iLPs (Figure 3D).

### 3.3. In Vitro Inhibition of P. falciparum Growth by iLPs Encapsulating Pyronaridine

**Figure 4:**
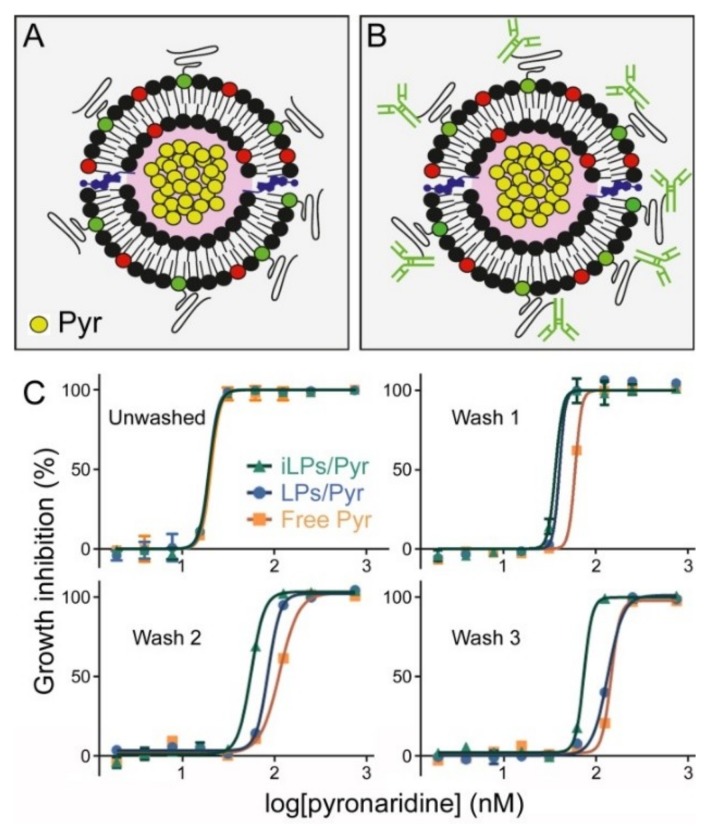
Pyronaridine growth inhibition assays in in vitro *P. falciparum* cultures. Scheme of pyronaridine-encapsulating (**A**) LP and (**B**) iLP. (**C**) Growth inhibition assays where pyronaridine was either (Unwashed) present for the whole 48 h of incubation or (Wash 1, 2, and 3) removed in 15 min-spaced washes.

In vitro growth inhibition assays of pyronaridine either in free form or encapsulated in LPs or iLPs (Figure 4A,B, respectively), and left in the culture for the whole 48 h of incubation, did not reveal significant differences among the three samples, which had a comparable IC_50_ around 19 nM (Figure 4C and Table 1). In another set of assays, after 15 min of incubation, pyronaridine was removed and replaced with fresh medium to better discern the effect of targeting and simulate drug removal in the blood circulation. In this case the activity of the encapsulated drug was improved relative to that of the free compound, a trend which was maintained when additional 15 min-spaced washing steps were done (Figure 4C). Although the IC_50_ of samples treated with iLP-encapsulated pyronaridine increased to ~74 nM after three washes (Table 1), the corresponding values for LP-encapsulated pyronaridine and for the free drug augmented significantly more (to ~135 and ~146 nM, respectively). The improvement in drug efficacy imparted by its encapsulation in iLPs was best observed for cultures incubated at 100 nM pyronaridine, where all three samples exhibited 100% growth inhibition before washing; after three washes; however, only the iLP-encapsulated drug maintained 100% activity at this concentration, whereas free and LP-encapsulated pyronaridine had only 5% and 20% activity, respectively. The reduced IC_50_ upon encapsulation in plain LPs vs. free drug is in agreement with the observed interaction with RBCs of LPs lacking anti-GPA targeting (Figure 3B).

### 3.4. Simultaneous Encapsulation in Liposomes of Pyronaridine and DB

**Figure 5:**
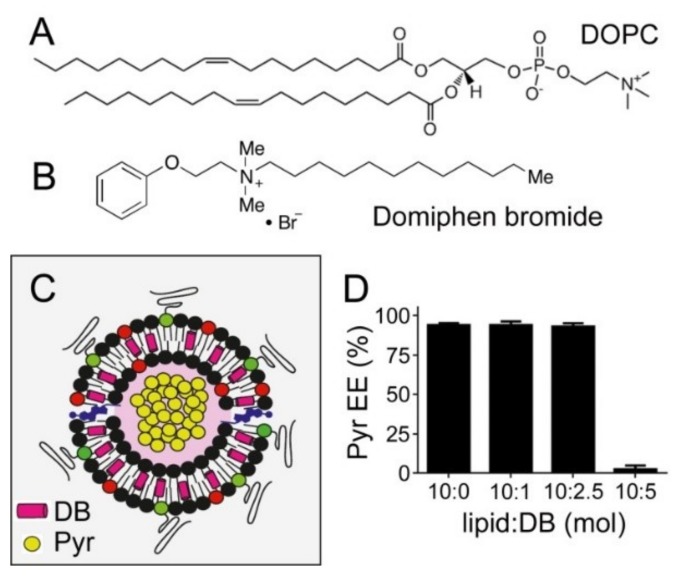
Co-encapsulation in liposomes of pyronaridine and DB. (**A**,**B**) Chemical structures of DOPC and DB. (**C**) Scheme of pyronaridine and DB co-encapsulation. (**D**) Encapsulation efficiency (EE) of pyronaridine in liposomes (5 mM lipid, ca. 10:1 lipid:pyronaridine initial ratio) at different lipid:DB ratios.

**Figure 6:**
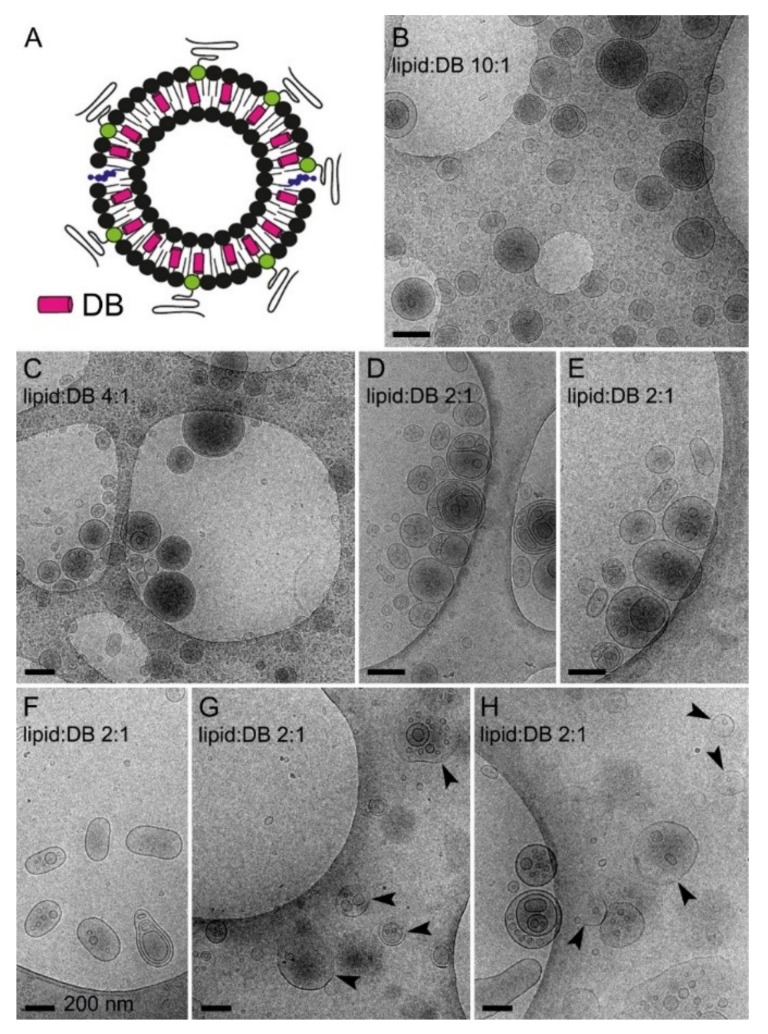
CryoTEM analysis. (**A**) Scheme of the DB-encapsulating liposome. (**B**–**H**) Images of liposomes containing different lipid:DB molar ratios. Arrowheads indicate deteriorated liposomes. Scale bars: 200 nm.

The chemical structure of DB, with a long saturated carbon tail similar in dimensions to that of phospholipids (Figure 5A,B), suggested that it could be incorporated into the lipid bilayer of LP membranes without significantly altering their properties in terms of structure and the capacity to encapsulate pyronaridine (Figure 5C). The incorporation of DB in liposomes was independent of the presence of a pH gradient (Figure S5). DOPC-based liposomes, prepared incorporating increasing DB amounts in their formulations, were loaded with pyronaridine at a lipid:pyronaridine molar ratio of ca. 10:1 using the pH gradient method described above. High pyronaridine encapsulation efficiencies (>95%) were observed up to 2.5 mM DB (4:1 lipid:DB ratio; Figure 5D and Figure S6), but these dropped to ca. 2% when reducing the lipid:DB ratio to 2:1 (Figure 5D). This result indicated that above a certain DB content the liposome lipid bilayer becomes destabilized and is no longer capable of sustaining the transmembrane pH drop required to encapsulate pyronaridine. CryoTEM examination confirmed that at lipid:DB ratios down to 4:1, liposomes appeared well formed with an intact membrane (Figure 6B,C). Although, at the lipid:DB ratio 2:1, liposomes formed correctly according to fluorescence microscopy and dynamic light scattering analysis (Figure S2B,C), lipid bilayer alterations were evident in cryoTEM images, such as oblong shapes and deteriorated membranes (Figure 6D–H).

### 3.5. In Vitro Inhibition of P. falciparum Growth by iLPs Encapsulating DB

In vitro growth inhibition assays of DB, either in free form or encapsulated in LPs or iLPs, showed, in washed samples, a significant improvement in the activity of iLP-encapsulated DB relative to the free drug (Table 2). The IC_50_ of DB in in vitro *P. falciparum* cultures (~1 µM) was comparable to that previously reported for fosmidomycin (0.29–0.37 µM [16]). However, as a consequence of the limit in the maximum DB content that can be incorporated in liposomes without impairing their stability, this relatively high IC_50_ required the addition to the cultures of DB-encapsulating iLPs in quantities inducing erythrocyte agglutination (Figure S7). This result led us to discard DB for targeted immunoliposome approaches. The antimalarial drug atovaquone, with an in vitro IC_50_ for *P. falciparum* in the low nM range [35], comparable to that of pyronaridine, was selected for the proof-of-concept assay of drug co-encapsulation in targeted iLPs. The dimensions and molecular shape of atovaquone, similar to those of cholesterol (the lengths of both molecules are 14 and 15 C–C bonds, respectively), suggested that it could comfortably fit into the lipid bilayer of a liposome.

### 3.6. In Vitro Inhibition of P. falciparum Growth by iLPs Co-Encapsulating Pyronaridine and Atovaquone

**Figure 7:**
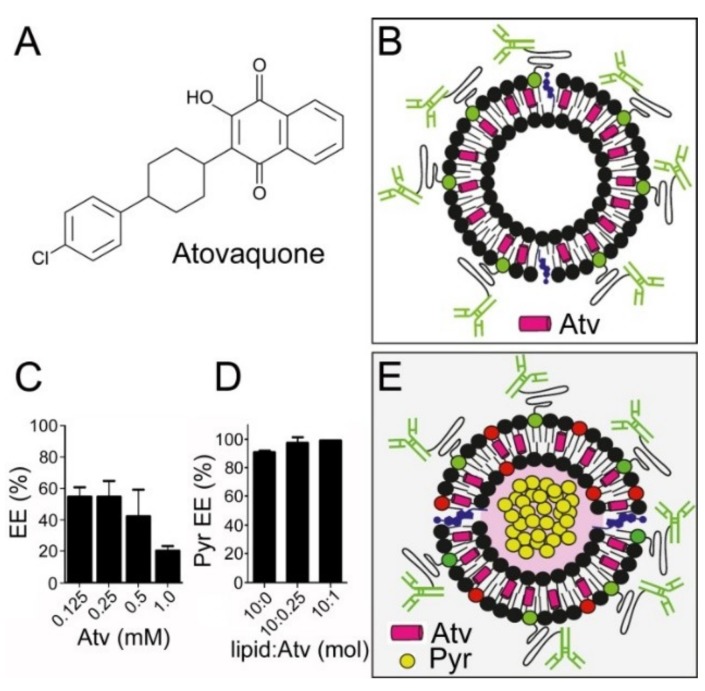
iLPs co-encapsulating pyronaridine (Pyr) and atovaquone (Atv). (**A**) Atovaquone structure. (**B**) Scheme of the atovaquone-encapsulating immunoliposome. (**C**) Atovaquone encapsulation efficiency (EE) at different initial drug concentrations (10 mM lipid). (**D**) Encapsulation efficiency of pyronaridine in liposomes (5 mM lipid, ca. 10:1 lipid:pyronaridine initial ratio) at different lipid:atovaquone molar ratios. (**E**) Scheme of the immunoliposome encapsulating pyronaridine and atovaquone.

Atovaquone (Figure 7A) could be loaded in POPC-based liposomes (Figure 7B) with a maximum encapsulation efficiency of about 50% (Figure 7C), although, because of its small IC_50_, this yield permitted the incorporation of the drug amounts required to perform in vitro activity assays without risk of inducing agglutination. As described above for pyronaridine and DB, the activity in three-times-washed samples of atovaquone improved following its encapsulation in targeted liposomes, with respective IC_50_ values for the free drug and iLP-drug of 77.6 and 18.6 nM (57.6–104.6 and 13.1–26.4 95% confidence intervals, respectively). Liposomes, prepared incorporating increasing atovaquone amounts in their formulations, were loaded with pyronaridine at a lipid:pyronaridine molar ratio of 10:1 using the pH gradient method described above. High pyronaridine encapsulation efficiencies (>90%) were observed up to a lipid:atovaquone ratio of 10:1 (Figure 7D), far above that required to obtain in the in vitro *P. falciparum* cultures an inhibitory concentration of immunoliposomized atovaquone. When immunoliposomes were prepared co-encapsulating pyronaridine and atovaquone (containing 10:0.5 lipid:atovaquone molar ratio; Figure 7E), their combined in vitro activity was significantly higher than when both drugs were incorporated into the cultures in their free forms. At drug concentrations that, in three-wash co-encapsulated immunoliposomized samples, induced a 50% of growth inhibition, namely 43.1 nM atovaquone and 137.2 nM pyronaridine (29.6–62.7 and 90.7–207.4 95% confidence intervals, respectively), the same amounts of free compounds did not reach 1% inhibition (Table 3). The higher amount needed of each encapsulated individual drug to reach 50% growth inhibition, when used in combination relative to when used alone, was also observed for the free compounds, which required 116 nM atovaquone and 379 nM pyronaridine in three-times-washed samples.

## 4. Discussion

It is generally accepted that to achieve malaria eradication a combination of weapons will be needed [43], including the improvement of existing approaches and the development of new ones [44], with drug therapy remaining the mainstay of treatment and prevention [45,46], and nanotechnology being able to provide innovative useful tools [47]. When drugs are administered together in the same nanostructure, they will be eliminated from the organism simultaneously with their carrier, thus maintaining constant relative ratios throughout the treatment, which will likely end up in improved pharmacokinetics. Co-nanoencapsulation of the antimalarials quinine and curcumin in polysorbate-coated polymeric nanoparticles inhibited *P. falciparum* growth in vitro to a slightly larger extent than identical amounts of the unencapsulated drugs [48]. In an in vivo study with the murine malaria species *Plasmodium berghei*, curcumin-artesunate co-entrapped in poly(D,L-lactic-*co*-glycolic acid) nanoparticles resulted in significant parasitemia reduction and increased mice survival when compared with the free compounds [49].

Liposomes are particularly adept structures for nanosized combination delivery systems because they allow the encapsulation of hydrophobic molecules in their lipid bilayer and of water-soluble compounds in their lumen, thus being a potentially interesting platform for combination therapies where lipophilic and hydrophilic drugs are delivered together in the same nanocarrier. The in vitro activity in *P. falciparum* cultures of pyronaridine and atovaquone co-encapsulated in immunoliposomes was significantly increased relative to the same amounts of both drugs in their free form. Because in the cultures there is no preferential elimination of the non-encapsulated compounds, this improvement in activity must be a consequence of the specific targeting of iLPs to pRBCs imparted by anti-glycophorin A antibodies. When DOPE-Rho-containing, GPA-targeted iLPs were loaded with the hydrophilic fluorescent dye pyranine and added to RBCs, both fluorescent labels were found in the cells [32]. The observed pattern suggested that, upon interaction, cells and liposomes eventually merged and, in this process, the liposomal lipid bilayer (and any lipophilic drug present therein) was incorporated into the cell membrane system, whereas the hydrophilic luminal content of the liposomes entered the cytosol. The growth inhibition assays performed in this work have been done in parasite cultures synchronized at early ring stages and with the drugs present for only 45 min. The observed improved activity of immunoliposomized drugs indicates their rapid cell binding and efficient internalization. A second benefit of targeting glycophorin A, an antigen present on all RBCs, is that, in this way, drugs can be efficiently delivered to early parasite ring stages, which do not expose *Plasmodium*-specific antigens, and to gametocytes. The finding that atovaquone and pyronaridine used together are less active against the malaria parasite than when used alone suggests that this particular drug combination is antagonistic. It is well known that, in combination therapies, susceptibility to one drug may alter effectiveness of the other, leading to increased tolerance to both compounds [50].

Whereas current chemotherapeutic approaches against malaria are targeted at the asexual blood stages that are responsible for all symptoms and pathologies of the disease [51], the threat of resistance-driven treatment failure is prompting research oriented toward developing drugs that target the weakest spots in the *Plasmodium* life cycle represented by smaller populations, which are less likely to contain resistant individuals that would benefit from the removal of susceptible parasites [52]. The World Health Organization recommends, particularly in areas threatened by resistance of *P. falciparum* to artemisinin [7], the implementation of strategies to reduce transmission of the parasite forms that move between hosts [53,54]. Targeting gametocytes, the stage solely responsible for the transmission from the human to the mosquito vector, can ease exposure of the pathogen to drugs and reduce the likelihood of resistance emerging [54].

Isoprenoid precursor biosynthesis may be the only required essential function of the apicoplast during *Plasmodium* blood stage development [55,56], which highlights the potential importance of MEP pathway inhibitors for future malaria therapeutics. In enzymatic assays, DB had an IC_50_ ~169 nM for *P. vivax* IspD [24], significantly lower than the value reported here for *P. falciparum* living parasites (~1 µM). In in vitro activity assays done with the *P. falciparum* enzyme, the IspD inhibitor MMV008138 had an IC_50_ of ~44 nM, which increased to ~350 nM against asexual stages in in vitro cultures of the *P. falciparum* Dd2 strain [22,57], comparable to that of DB on the 3D7 strain used here. The fosmidomycin IC_50_ for DXR is one order of magnitude higher in *P. falciparum* cultures (~370 nM for the A2 strain) relative to the recombinant enzyme [16]. The results obtained here with DB indicate that erythrocyte agglutination might be induced by immunoliposomes encapsulating drugs with an IC_50_ for living parasites close to the µM range, which is the case for all MEP pathway inhibitors where data are available.

The inactivity of fosmidomycin in in vitro cultures of the apicoplastidic protist *Toxoplasma gondii* [57], which has DXR, is most likely due to the drug not reaching the enzyme location inside the living parasite. Because the molecular targets of MEP pathway inhibitors are inside the apicoplast, in order to exert their activity these drugs have to cross several lipid bilayers, namely those of the RBC, the parasitophorous vacuole, the intraerythrocytic *Plasmodium* parasite itself, and the apicoplast. As a result, the drug concentrations required to obtain antiparasitic activity increase significantly. The liposomal prototype presented here is designed to shuttle drugs only through the first one of these barriers. Future nanocarriers engineered to drive their contents also through some of the three other biological membranes should be able to further reduce the IC_50_ of MEP pathway inhibitors to make them a viable alternative for future clinically useful antimalarials.

The history of antimalarial chemotherapy clearly shows that resistance of the pathogen to every new chemical developed against it has, without exception, evolved after a few years following its deployment [58]. It will be; therefore, of the utmost importance to have efficient drug targeting strategies in place for the moment when new drugs appear, in order to administer them to intraerythrocytic malaria stages at sufficiently high local doses as to significantly reduce the evolution of resistant parasites. The straightforward consequence will be that the few antimalarials hitting the market will have a much more extended life before resistances evolve, which can give us an edge in the fight against *Plasmodium*. The nanocarrier developed here for a targeted combination therapy at the nanoscale can find an immediate application for the severe malaria cases that usually end up in health centers, where the intravenous administration of liposomal formulations is feasible. Future challenges that have to be dealt with include modifications of the nanocarrier in order to make it adequate for oral administration formulations used in non-complicated malaria, which will likely involve polymer-based nanocapsules instead of liposomes. A targeted nanomedicine like the one described above could be an essential tool for a future eradication scenario where the last few cases of hyper-resistant *Plasmodium* strains will have to be wiped off with extremely high local doses of combinations of future new drugs.

## Figures and Tables

**Table 1:** Pyronaridine IC_50_ values (nM) in in vitro *P. falciparum* cultures. 95% confidence intervals are indicated in parentheses.

	No Wash	Wash 1	Wash 2	Wash 3
Free Pyr	20.6 (18.8–22.5)	57.2 (55.0–59.6)	113.6 (106.2–121.5)	146.4 (121.3–176.7)
LPs/Pyr	19.7 (17.7–21.9)	43.1 (40.0–46.3)	85.5 (80.7–90.7)	134.8 (128.1–141.9)
iLPs/Pyr	19.7 (18.5–21.0)	36.2 (17.4–75.2)	55.8 (52.4–59.4)	74.2 (62.0–88.8)

**Table 2:** DB IC_50_ values (µM) in in vitro *P. falciparum* cultures. 95% confidence intervals are indicated in parentheses. Agglutinated (p)RBCs were discarded in flow cytometry parasitemia determinations.

	No Wash	Wash 1	Wash 2	Wash 3
Free DB	1.1 (1.0–1.3)	4.1 (3.6–4.8)	6.0 (5.3–6.8)	8.3 (7.0–9.7)
LPs/DB	1.7 (1.5–1.9)	12.0 (9.6–15.0)	28.9 (18.5–45.3)	34.9 (16.2–74.9)
iLPs/DB	1.0 (0.8–1.2)	2.0 (1.7–2.2)	2.4 (2.1–2.7)	3.0 (2.7–3.4)

**Table 3:** Percentages of growth inhibition in in vitro *P. falciparum* cultures at the corresponding drug concentrations that induce a 50% inhibition in immunoliposomized samples.

	% Inhibition at Drug IC_50_ of Immunoliposome Three-Wash Sample		
	Immunoliposomized Drug	Liposomized Drug	Free Drug
Pyronaridine	50.0	16.2	2.0
Domiphen bromide	50.0	0.2	3.5
Atovaquone	50.0	17.3	0.0
Atovaquone/pyronaridine	50.0	28.4	0.8

## Supplementary Materials

The following are available online at https://www.mdpi.com/1999-4923/11/7/341/s1, Figure S1: Schematic representation of the preparation of the different liposomes used in this work, Figure S2: Characterization of DSPC- and DOPC-based liposomes encapsulating DB, Figure S3: Scheme of the chemical coupling of the anti-GPA antibody to a maleimide group-containing lipid, Figure S4: Pyronaridine encapsulation in DOPC-based liposomes in the presence of a pH gradient, Figure S5: DB incorporation in DOPC-based liposomes in the presence of a pH gradient, Figure S6: Pyronaridine encapsulation through the pH gradient method in DOPC-based liposomes containing in their membranes DB at a total lipid:DB ratio 4:1, Figure S7: Flow cytometry analysis of red blood cell agglutination induced by a suspension of immunoliposomes functionalized with anti-GPA antibodies and containing 25 μM DB.
