# Supplementary material for: An ImmunoPEGliposome for Targeted Antimalarial Combination Therapy at the Nanoscale
Source: Pharmaceutics. 2019 Jul 16;11(7):341. doi: 10.3390/pharmaceutics11070341 (PMC6680488; doi:10.3390/pharmaceutics11070341)
Supplement: Supplementary file 1 [file pharmaceutics-11-00341-s001.pdf]

pharmaceutics

An ImmunoPEGliposome for Targeted Antimalarial Combination Therapy at the Nanoscale

Arnau Biosca [1,2,3], Lorin Dirscherl [1,2,3], Ernest Moles [1,2,3], Santiago Imperial [3,4] and Xavier Fernàndez-Busquets[1,2,3,*]

[1] Nanomalaria Group, Institute for Bioengineering of Catalonia (IBEC), The Barcelona Institute of Science and Technology, Baldiri Reixac 10-12, ES-08028 Barcelona, Spain
[2] Barcelona Institute for Global Health (ISGlobal, Hospital Clínic-Universitat de Barcelona), Rosselló 149-153, ES-08036 Barcelona, Spain
[3] Nanoscience and Nanotechnology Institute (IN2UB), University of Barcelona, Martí i Franquès 1, ES-08028 Barcelona, Spain
[4] Department of Biochemistry and Molecular Biomedicine, University of Barcelona, Avda. Diagonal 643, ES-08028 Barcelona, Spain
* Author to whom correspondence should be addressed.

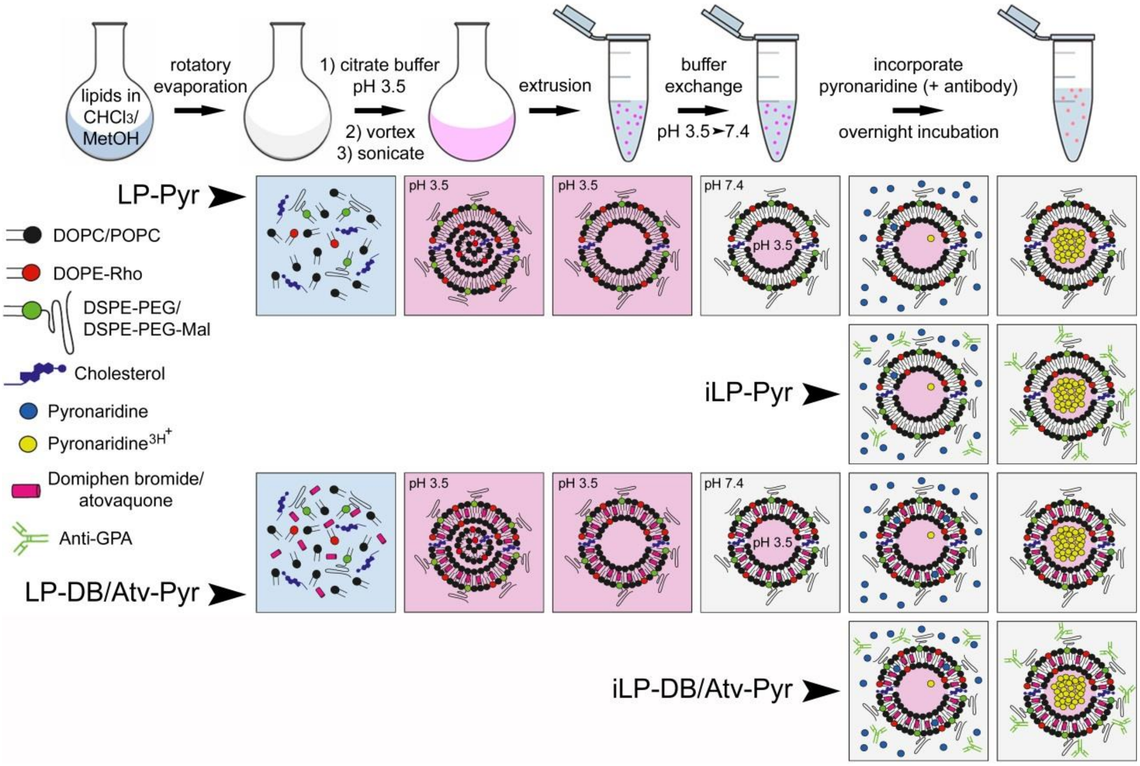

Figure 1. Schematic representation of the preparation of the different liposomes used in this work. For those liposomes not encapsulating pyronaridine, lipids were taken up in PBS instead of citrate buffer, and the buffer exchange and pyronaridine incorporation steps were omitted. LP: Liposome; iLP: Immunoliposome; Pyr: Pyronaridine; DB: Domiphen bromide; Atv: Atovaquone.

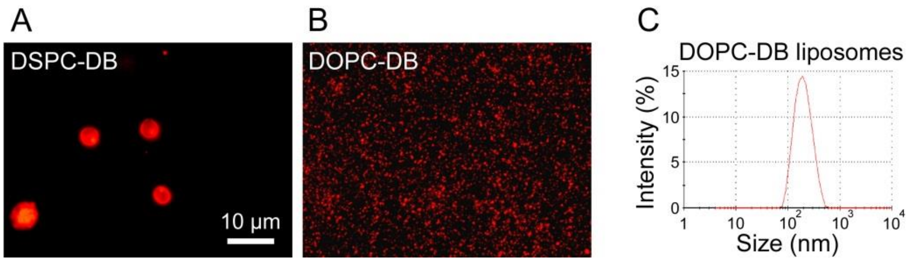

Figure 2. Characterization of DSPC- and DOPC-based liposomes encapsulating DB. (**A,B**) Fluorescence microscopy analysis of (**A**) DSPC- and (**B**) DOPC-based liposomes containing DOPE-Rho and encapsulating DB with a molar ratio total lipid:DB of 2:1. (**C**) Dynamic light scattering analysis of DOPC-DB liposomes.

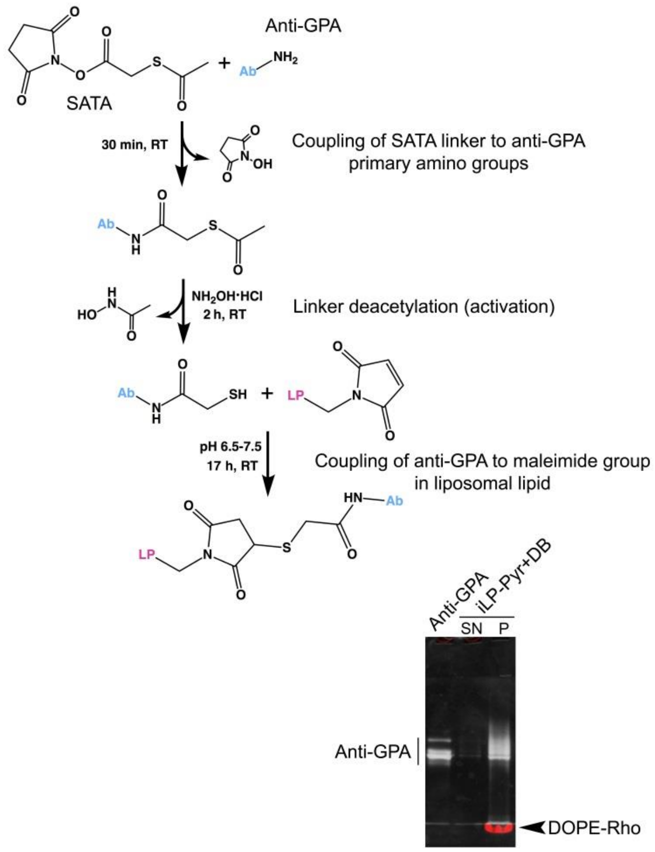

Figure 3. Scheme of the chemical coupling of the anti-GPA antibody (Ab) to a maleimide group-containing lipid (LP).

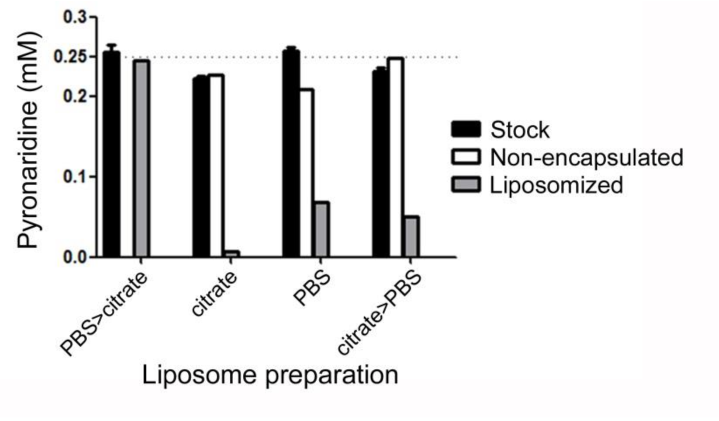

Figure 4. Pyronaridine encapsulation in DOPC-based liposomes in the presence of a pH gradient 7.4 outside → 3.5 inside (PBS > citrate), 3.5 outside → 7.4 inside (citrate > PBS), and in the absence of a pH gradient at pH 3.5 (citrate) and 7.4 (PBS).

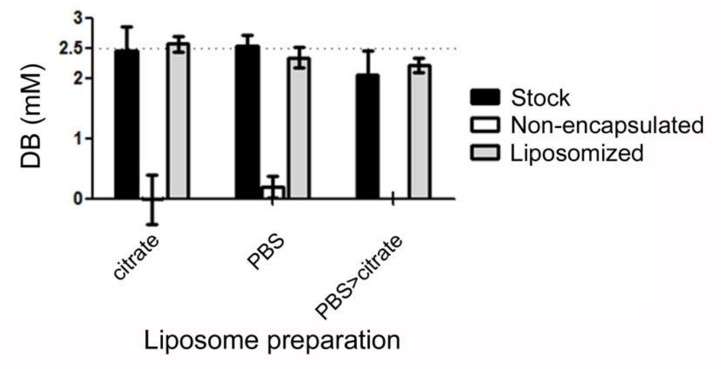

Figure 5. DB incorporation in DOPC-based liposomes in the presence of a pH gradient 7.4 outside → 3.5 inside (PBS > citrate), and in the absence of a pH gradient at pH 3.5 (citrate) and 7.4 (PBS).

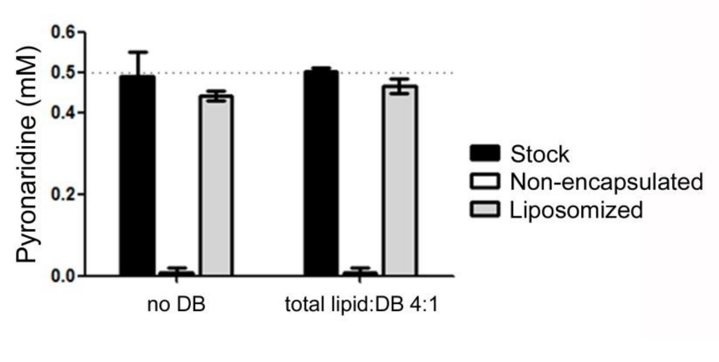

Figure 6. Pyronaridine encapsulation through the pH gradient method in DOPC-based liposomes containing DB at a total lipid:DB ratio of 4:1 in their membranes.

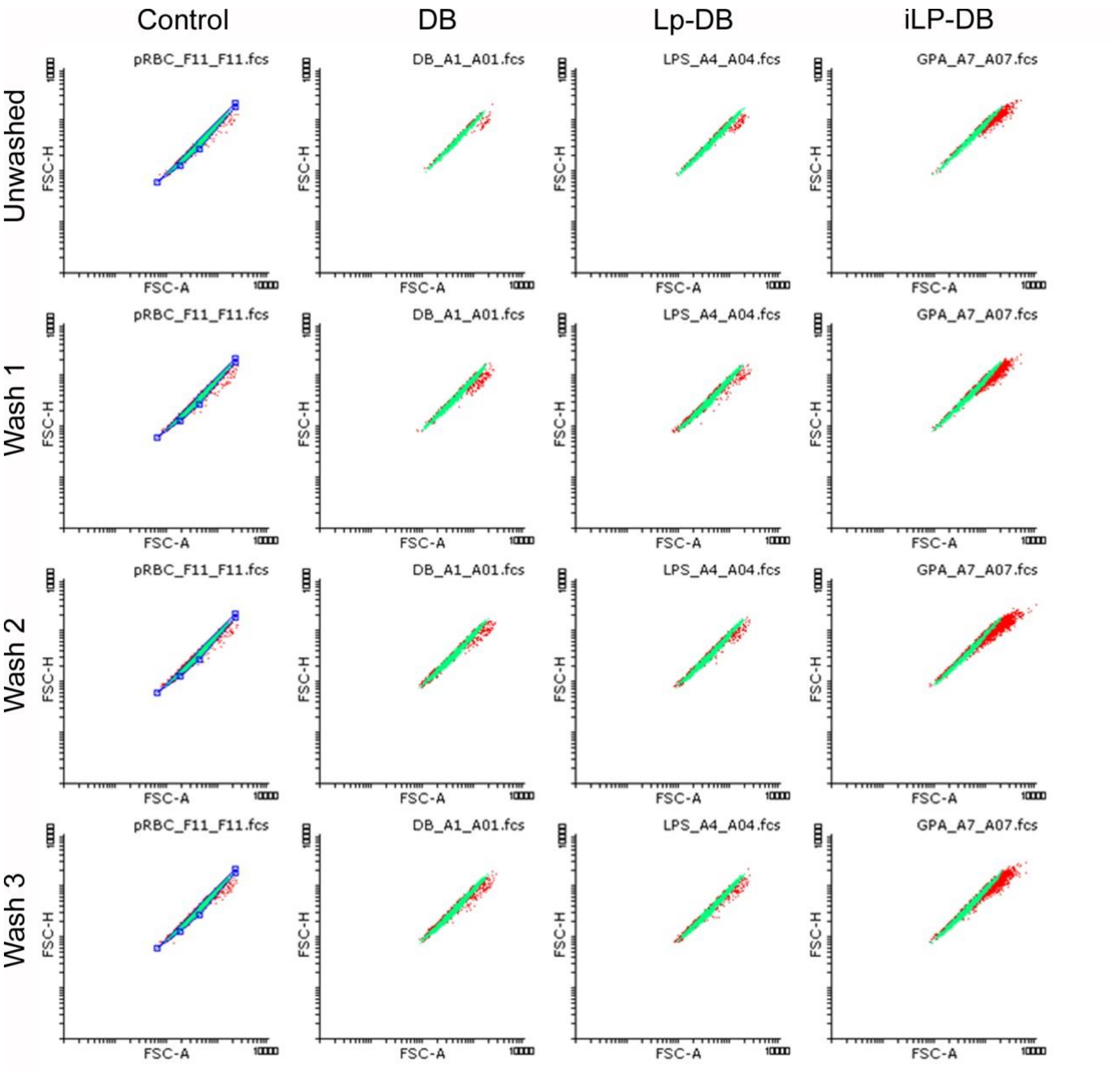

Figure 7. Flow cytometry analysis of red blood cell agglutination induced by a suspension of immunoliposomes functionalized with anti-GPA antibodies and containing 25 μM DB. Control samples containing no drug were used to gate the RBC agglutinates (shown in red). Free drug and plain liposome samples contained also 25 μM DB. RBC agglutinates increase significantly in immunoliposome samples, even after three washes.
